# Revision rate and postoperative volume development of chronic subdural hematomas after burr hole craniotomy in combination with tranexamic acid vs. surgery alone – a single-center propensity score-matched analysis

**DOI:** 10.1186/s12883-026-05003-3

**Published:** 2026-06-08

**Authors:** Magnus Scheer, Hanno Witte, Paulina Guenzerodt, Vanessa Beuchel, Uwe Max Mauer, Chris Schulz

**Affiliations:** 1 Department of Neurosurgery, German Armed Forces Hospital Ulm, Oberer Eselsberg 40, 89081 Ulm, Germany; 2 Department of Internal Medicine, Bundeswehr Hospital Ulm, 89081 Ulm, Germany; 3 Department of Neurosurgery, University Hospital Augsburg, Augsburg, Germany; 4 Department of Neurosurgery, German Armed Forces Hospital Westerstede, Lange Str. 38, 26655 Westerstede, Germany

**Keywords:** Tranexamic acid, Chronic subdural hematoma, Recurrence after surgery, Propensity score matching

## Abstract

**Background:**

Chronic subdural hematoma (cSDH) is a common intracranial hemorrhage in elderly patients and is associated with substantial postoperative recurrence rates. Tranexamic acid (TXA) has been proposed as an adjuvant therapy to reduce recurrence by targeting hyperfibrinolysis; however, its efficacy and impact on hematoma volume evolution remain controversial.

**Methods:**

We performed a retrospective cohort study of adult patients who underwent burr-hole evacuation with subdural drainage for cSDH at a single neurosurgical center between 2012 and 2024. Patients receiving postoperative TXA within 48 h for at least 30 days were compared with patients treated surgically without TXA. Propensity score matching (1:1) was applied to balance baseline characteristics. The primary outcome was revision surgery for recurrent cSDH within 3 months. Secondary outcomes included postoperative hematoma volume evolution and all-cause mortality.

**Results:**

After matching, 73 patients were included in each group with well-balanced baseline characteristics. Revision surgery within 90 days occurred less frequently in the TXA group compared with controls (8.2% vs. 19.2%; OR 0.40, 95% CI 0.14–1.12; *p* = 0.042), although the confidence interval marginally crossed unity, indicating limited precision. Median time to revision was 8 days in the TXA group and 11 days in the control group. Mortality was numerically lower in the TXA group, with no deaths observed, compared with one death (1.4%) in the control group. Preoperative, postoperative, and one-month follow-up hematoma volumes were comparable between groups, and no significant difference in absolute volume reduction was detected.

**Conclusion:**

Postoperative adjuvant TXA therapy after surgical evacuation of cSDH was associated with a lower rate of recurrence requiring revision surgery, without an observed increase in mortality; however, the confidence interval marginally crossed unity, and the findings should be regarded as hypothesis-generating. TXA did not significantly influence short-term hematoma volume reduction. Prospective randomized studies are needed to confirm these findings and define optimal dosing strategies.

## Introduction

Chronic subdural hematoma (cSDH) represents one of the most common traumatic intracranial hemorrhages in Western industrialized countries [28]. It is defined as a persistent accumulation of hemorrhagic to serous fluid within the subdural space, typically resulting from a mild traumatic brain injury that occurred several weeks earlier. The reported incidence (1.7–20.6 per 100,000 inhabitants) increases significantly with age, posing a growing challenge in the context of ongoing demographic change [4].

The pathophysiology of cSDH is complex and cannot be explained solely by the accumulation of blood following rupture of bridging veins. Rather, the underlying mechanism is thought to involve injury to the dural border cell layer, a highly specialized connective tissue cell layer located between the dura mater and the arachnoid mater. Disruption of this layer initiates a local inflammatory response, which, through the induction of angiogenesis, leads to the formation of pathological, thin-walled capillaries that are prone to rupture and cause recurrent microhemorrhages. In parallel, plasminogen-activated hyperfibrinolysis of the hematoma content—considered an acute-phase reaction—impairs physiological clot formation [5, 9]. This results in a self-perpetuating cycle of cellular proliferation, angiogenesis, hyperfibrinolysis, and recurrent bleeding, ultimately driving the progressive enlargement of the hematoma [3, 21].

Treatment strategies for cSDH range from conservative management in asymptomatic patients with small hematomas (wait-and-watch strategy) to surgical decompression in cases of radiologically confirmed brain compression accompanied by neurological deficits [11]. Surgical approaches include burr-hole trephination or craniotomy with drainage [10]. Regardless of the surgical technique employed, relatively high recurrence rates (2.3%–38.7%) requiring revision surgery have been reported [17, 22]. In light of these recurrence rates, adjunctive conservative treatment strategies have gained increasing attention [8], with particular focus on targeting hyperfibrinolysis. Several studies investigating perioperative pharmacological therapy have reported promising results with tranexamic acid (TXA) [6, 12–14, 18, 23, 26, 27, 30, 31]. As a specific antifibrinolytic agent, TXA inhibits plasminogen activation and thereby suppresses hyperfibrinolysis within the hematoma, potentially preventing re-expansion of cSDH through the reduction of recurrent microbleeding [20]. However, this therapeutic approach remains controversial, as several studies have also reported no significant reduction in recurrence rates or even increased mortality associated with adjuvant TXA therapy [2, 24].

The aim of the present study was to evaluate the efficacy of tranexamic acid with regard to reducing postoperative recurrence rates as well as postoperative hematoma volume reduction following surgical evacuation of chronic subdural hematomas.

## Methods and materials

### Study population and inclusion criteria

We retrospectively analyzed patients treated for chronic subdural hematoma (cSDH) at the Department of Neurosurgery, German Armed Forces Hospital in Ulm, between 2012 and 2024. All patients aged ≥ 18 years who underwent unilateral or bilateral hematoma evacuation via burr-hole craniotomy with placement of a subdural drain during this period were eligible for inclusion. Patients with incomplete clinical data, including missing or incomplete cross-sectional imaging, as well as patients who developed an acute subdural hematoma due to immediate postoperative rebleeding, were excluded.

Bilateral chronic subdural hematomas were treated as a single case per patient. Each patient was counted once in the analysis, regardless of unilateral or bilateral hematoma presentation.

Two cohorts were defined: an intervention group comprising patients who received oral tranexamic acid (TXA), initiated within two days after surgical evacuation of cSDH and continued for a minimum of 30 days, without any preceding intravenous loading dose or transition regimen, and a control group consisting of patients who underwent surgical treatment without adjuvant therapy.

TXA was introduced at our institution in 2012 as an adjunctive option for recurrence prophylaxis based on emerging evidence in the literature. Throughout the study period, surgical and postoperative management of cSDH remained unchanged.

TXA use was at the discretion of the treating neurosurgeon and was not governed by a predefined institutional protocol. A total of ten neurosurgeons were involved in surgical management and postoperative treatment decisions during the study period. The specific rationale underlying individual decisions to administer or withhold TXA was not systematically documented and can no longer be reconstructed from the available records; the absence of predefined treatment criteria therefore represents a potential source of indication bias. The only contraindications were known hypersensitivity and active thromboembolic disease. No standardized dose adjustments were applied based on renal function. Thromboelastography was not used.

This cohort study was approved by the Ethics Committee of Ulm University on October 10, 2023 (approval number: 311/23) and was conducted in accordance with the Declaration of Helsinki as revised in 2008.

## Study outcomes

The primary outcome was the need for revision surgery due to recurrent hematoma within three months following the initial operation.

Secondary outcomes included postoperative volume evolution of the residual hematoma in the immediate postoperative period and at one-month follow-up, as well as overall mortality in both groups during a total observation period of three months.

Hematoma volumes were determined by manual volumetric segmentation on axial cranial computed tomography (CT) images using the Visage 7 diagnostic imaging platform (Visage Imaging GmbH, Berlin, Germany). The hematoma boundary was manually delineated on each axial slice using the freehand 3D region-of-interest (ROI) tool. Slice-by-slice contours were automatically integrated across all relevant slices to compute the total hematoma volume in milliliters.

### Statistical analysis

Statistical analyses were performed using R (R Foundation for Statistical Computing, Vienna, Austria) with the packages *MatchIt*,* tableone*,* pROC*,* effsize*,* dplyr*,* tidyr* and *ggplot2*. To account for baseline imbalances between patients treated with tranexamic acid (TXA) and those without TXA, we applied propensity score matching using the *MatchIt* package. The propensity score was estimated in a logistic regression model with TXA exposure as the dependent variable and age, hematoma architecture classified according to the Nakaguchi classification [19], and pre-existing anticoagulation as covariates. Patients were then matched in a 1:1 ratio using optimal matching based on the logit of the propensity score. Covariate balance before and after matching was assessed with the *tableone* package by comparing summary statistics and calculating standardized mean differences for all baseline variables. Group comparisons between TXA and no-TXA patients in the matched cohort were performed using *CreateTableOne*, which employs χ² tests or Fisher’s exact tests for categorical variables and Student’s t tests or Wilcoxon rank-sum tests for continuous variables, as appropriate.

The primary outcome was the need for surgical revision. The association between TXA use and revision surgery was evaluated in the matched sample using contingency tables and Fisher’s exact test. Additionally, a sensitivity analysis was performed after exclusion of all patients receiving oral anticoagulation therapy to assess the robustness of the primary findings. Recurrence rates were compared using Fisher’s exact test, and odds ratios with 95% confidence intervals were calculated. This analysis was exploratory and not powered for subgroup comparisons. To determine an optimal hematoma volume threshold for predicting revision, receiver operating characteristic (ROC) analysis was conducted with the *pROC* package. The ROC analysis was performed using postoperative hematoma volumes measured on the first routine postoperative cranial CT scan. The analysis was conducted in the overall cohort prior to propensity score matching, with pooled inclusion of TXA-treated and non-TXA-treated patients, using revision (yes/no) as the binary outcome, in order to determine a general postoperative hematoma volume threshold associated with revision surgery. The optimal cut-off was derived from the Youden index. To quantify patients with a hematoma volume exceeding the ROC-based cut-off, a dichotomous event variable was created. Group comparisons were performed using a two-sided Fisher’s exact test due to low event rates. Changes in hematoma volume over time (VolDiff: preoperative to postoperative change; VolDiff2: postoperative to 1-month follow-up change) were summarized by means and standard deviations within each group and compared between TXA and no-TXA patients using both Student’s t tests and Wilcoxon rank-sum tests. Effect sizes for volume reduction were quantified using Cohen’s d with the *effsize* package. Unless stated otherwise, all tests were two-sided and a p-value < 0.05 was considered statistically significant.

## Results

### Patient characteristics

**Fig. 1:**
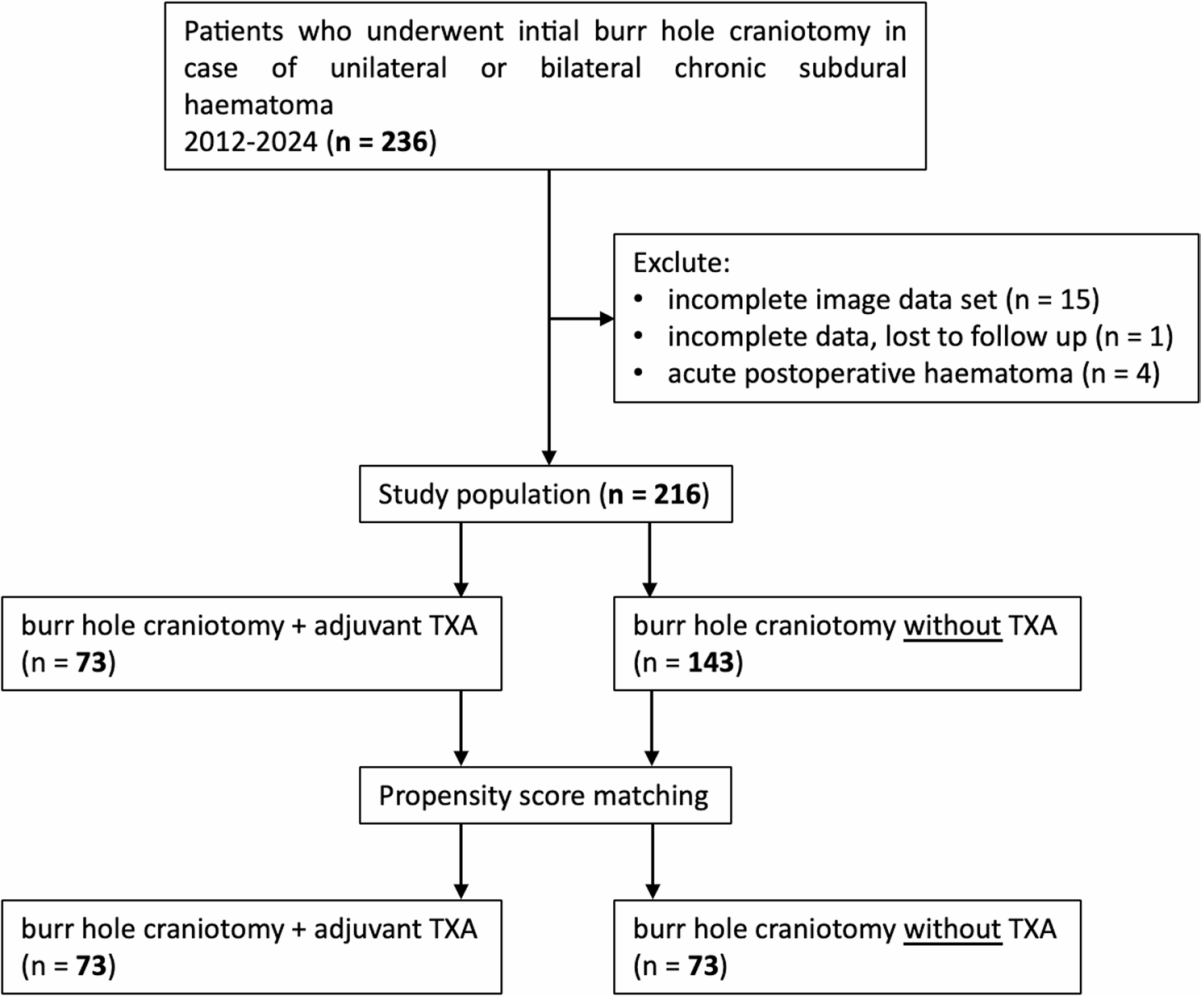
Study design, TXA: tranexamic acid

Between January 2012 and October 2024, a total of 236 patients with chronic subdural hematoma (cSDH) were treated by burr-hole trephination with placement of a subdural drain at the Department of Neurosurgery, Bundeswehr Hospital Ulm. Of these, 216 patients met the inclusion criteria for the retrospective cohort analysis. Within the study population, 73 patients received adjuvant oral therapy with tranexamic acid (TXA), initiated within 48 h after surgery and continued for a minimum of 30 days. The remaining 143 patients underwent standard treatment consisting of surgical hematoma evacuation without additional therapy (Fig. 1).

In the unmatched cohort, significant differences between groups were observed, particularly with respect to the use of direct oral anticoagulants (DOACs) (35 vs. 3 patients, *p* < 0.001). After propensity score matching, 73 patients remained in each group, with adequate balance of the selected covariates. Specifically, the use of DOACs (3 vs. 3, *p* = 1.000), Nakaguchi classification (type I: 38 vs. 38; type II: 7 vs. 5; type III: 4 vs. 4; type IV: 24 vs. 26; *p* = 0.937), history of hypertension (34 vs. 34, *p* = 1.000), and use of platelet aggregation inhibitors (13 vs. 13, *p* = 1.000) were evenly distributed between the groups. No statistically significant differences in baseline characteristics were observed after matching.

Following propensity score matching, age and sex distributions were comparable between the TXA and control groups. In the TXA group, 50 patients were male (68.5%) and 23 were female (31.5%), whereas in the non-TXA group, 59 patients were male (80.8%) and 14 were female (19.2%); the difference in sex distribution was not statistically significant (*p* = 0.128). Mean age was 72.90 years in the TXA group and 73.29 years in the control group (*p* = 0.855), indicating successful balancing of these baseline characteristics through matching. (Table 1)

**Table 1:** Patient characteristics and results pre- and post-matching, CKD, chronic kidney disease; PAI, platelet aggregation inhibition, TXA, tranexamic acid, Preop, preoperativ

Characteristics	All patients			Matched pairs		
	No TXA (*n* = 143)	TXA (*n* = 73)	*P* value	No TXA (*n* = 73)	TXA (*n* = 73)	*P* value
Sex: *n* (%)						
m	112(78.3)	50 (68.5)	0.158	59 (80.8)	50 (68.5)	0.128
f	31 (21.7)	23 (31.5)		14 (19.2)	23 (31.5)	
Age (median)	74.99	72.90	0.263	73.29	72.90	0.855
Nakaguchi classification: n (%)						
1	81 (56.6)	38 (52.1)	0.117	38 (52.1)	38 (52.1)	0.937
2	12 (8.4)	5 (6.8)		7 (9.6)	5 (6.8)	
3	18 (12.6)	4 (5.5)		4 (5.5)	4 (5.5)	
4	32 (22.4)	26 (35.6)		24 (32.9)	26 (35.6)	
CKD: n (%)						
1	25 (38.5)	15 (22.7)	0.197	25 (39.7)	15 (22.7)	0.172
2	20 (30.8)	32 (48.5)		19 (30.2)	32 (48.5)	
3a	13 (20.0)	11 (16.7)		12 (19.0)	11 (16.7)	
3b	4 (6.2)	6 (9.1)		4 (6.3)	6 (9.1)	
4	3 (4.6)	2 (3.0)		3 (4.8)	2 (3.0)	
Anticoagulation: n (%)	35 (24.5)	3 (4.1)	< 0.001	3 (4.1)	3 (4.1)	1.000
PAI: n(%)	22 (30.1)	13 (17.8)	0.121	13 (17.8)	13 (17.8)	1.000
Diabetes mellitus: n (%)	26 (18.2)	7 (9.6)	0.144	14 (19.2)	7 (9.6)	0.157
Hypertension: n (%)	82 (57.3)	34 (46.6)	0.175	34 (46.6)	34 (46.6)	1.000
Seizure: n (%)	7 (4.9)	1 (1.4)	0.359	3 (4.1)	1 (1.4)	0.612
Localization: n (%)						
Bilateral	23 (16.1)	14 (19.2)	0.743	12 (16.4)	14 (19.2)	0.790
Left	68 (47.6)	31 (42.5)		35 (47.9)	31 (42.5)	
Right	52 (36.4)	28 (38.4)		26 (35.6)	28 (38.4)	
Hematoma volume: ml (median)						
preoperative	100	97	0.840	94	104	0.949
postoperative	60	58	0.881	57	60	0.637
1 month postoperative	35	20	0.107	25	20	0.232
*Decrease* preop to 1 month postsurgical	70	74	0.381	68	85	0.361
Revision: n (%)	23 (16.1)	6 (8.2)	0.178	14 (19.2)	6 (8.2)	0.042
Complications: n (%)						
Death events	5 (3.5)	0 (0)		1 (1.4)	0 (0)	0.0175**
Neurologic impairment*	1 (0)	0 (0)		0 (0)	0 (0)	
Surgery related	1 (0)	0 (0)		0 (0)	0 (0)	
Others	3 (2.1)	0 (0)		1 (1.4)	0 (0)	

* new after surgery ** overall complications

In the TXA group, the mean daily dose of tranexamic acid was 1,226 mg administered for a minimum duration of 30 days (range: 500–2,000 mg). Forty-five patients (61.6%) received a total daily dose of 1,500 mg, divided into two or three administrations. Fourteen patients (19.2%) received 500 mg, four patients (5.5%) received 750 mg, eight patients (11.0%) received 1,000 mg, and two patients (2.7%) received 2,000 mg as the total daily dose.

### Outcome measures

#### Primary outcome

**Fig. 2:**
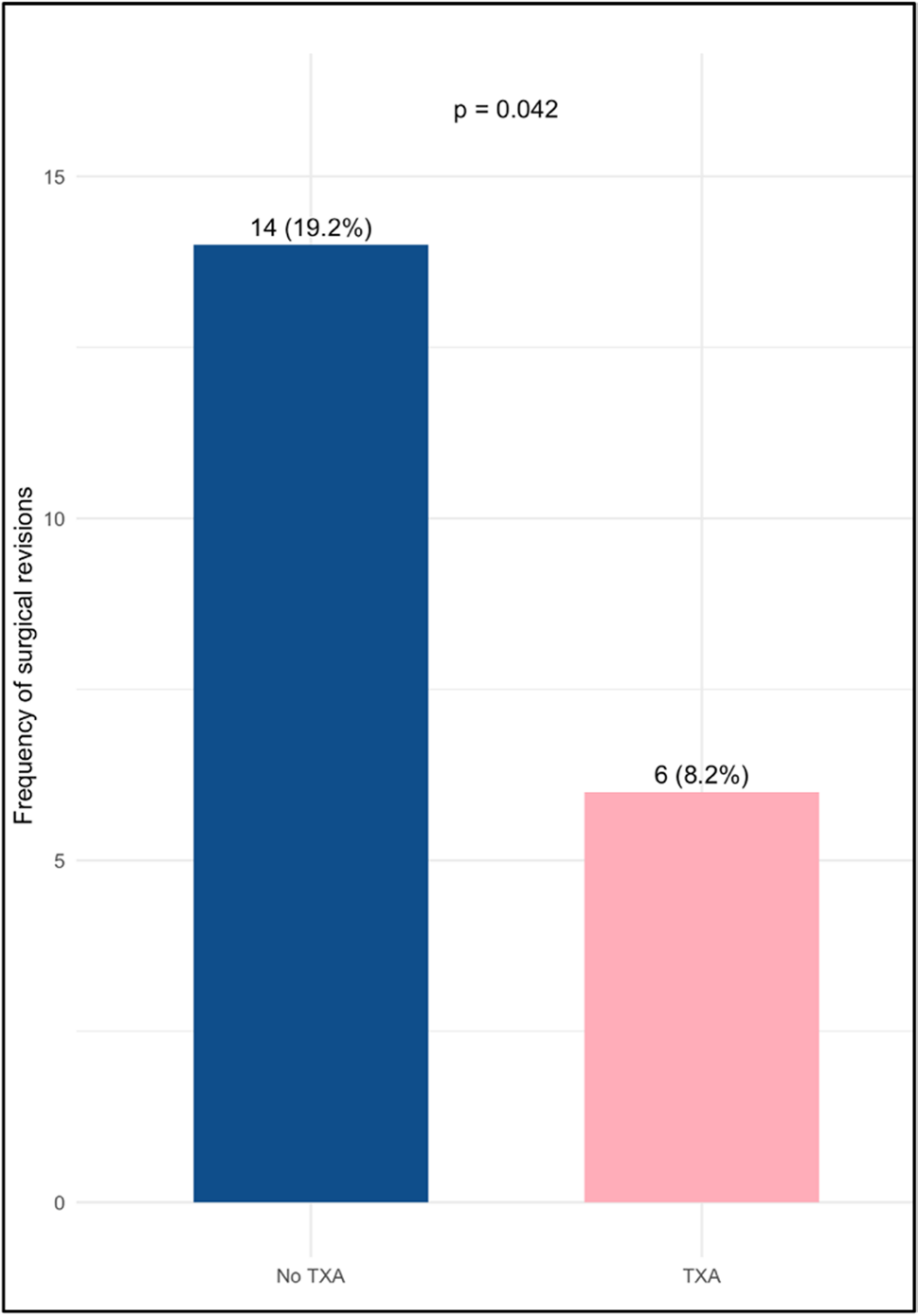
Rate of revision in each group, TXA: tranexamic acid

**Fig. 3:**
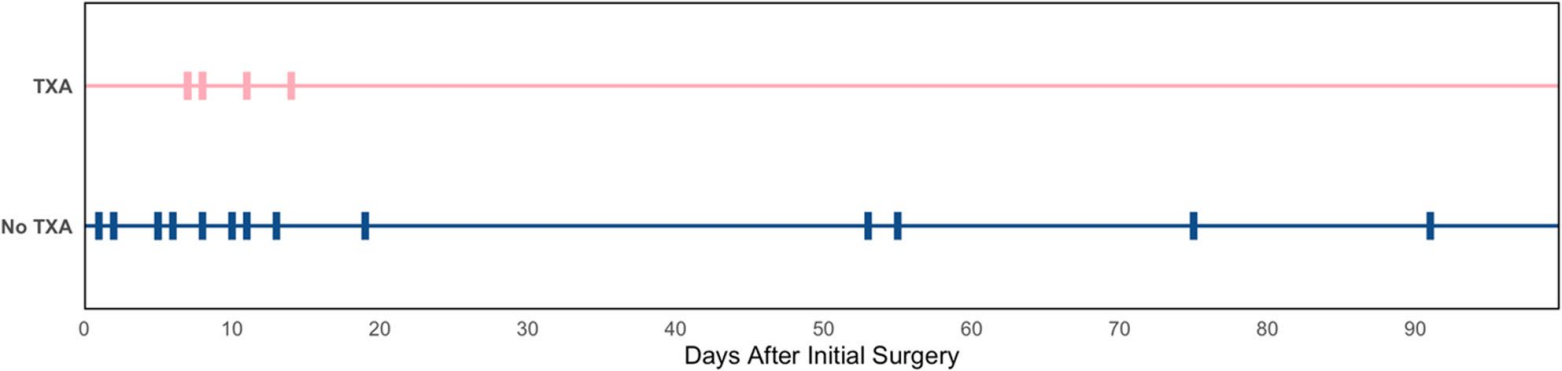
Time interval between initial surgery and revision in days, depending on treatment arm, TXA: tranexamic acid

Before propensity score matching, the 90-day revision surgery rate was higher in the control group than in the TXA group (16.1% vs. 8.2%; OR = 0.452; 95% CI: 0.175–1.65), without reaching statistical significance (*p* = 0.178). (Fig. 2) After matching, the revision rate was higher in the control group compared with the TXA group (19.2% vs. 8.2%; OR = 0.400; 95% CI: 0.143–1.118; *p* = 0.042). In the analysis after exclusion of all patients receiving oral anticoagulation therapy, recurrence occurred in 7.1% of TXA-treated patients and in 19.4% of controls (OR 0.37; 95% CI 0.10–1.12; *p* = 0.068). Median time to revision surgery was 11 days (range: 1–91 days) in the control group and 8 days (range: 8–14 days) in the TXA group (Fig. 3). Among TXA-treated patients requiring revision surgery, two-thirds received daily TXA doses below 1,500 mg (500 mg: 16.7%; 750 mg: 50%). Two of six patients (33.3%) received a total daily dose of 1,500 mg.

In the overall cohort prior to propensity score matching, four patients experienced multiple recurrences requiring repeated revision surgery: three patients underwent two revision procedures each, and one patient required three revisions. All four patients belonged to the non-TXA group and were not included in the propensity score–matched cohort. In the matched cohort, all recurrence events were single revisions in both groups.

## Secondary outcomes

Before matching, five deaths (3.5%) occurred in the control group within 90 days, whereas no deaths were observed in the TXA group. After matching, one death (1.4%) was recorded in the control group during the follow-up period.

After matching, median preoperative hematoma volumes were comparable between groups (TXA: 104 mL; control: 94 mL; *p* = 0.949). No significant differences were observed in residual hematoma volume immediately postoperatively (60 mL vs. 57 mL; *p* = 0.637) or at one-month follow-up (20 mL vs. 25 mL; *p* = 0.232).

**Fig. 4:**
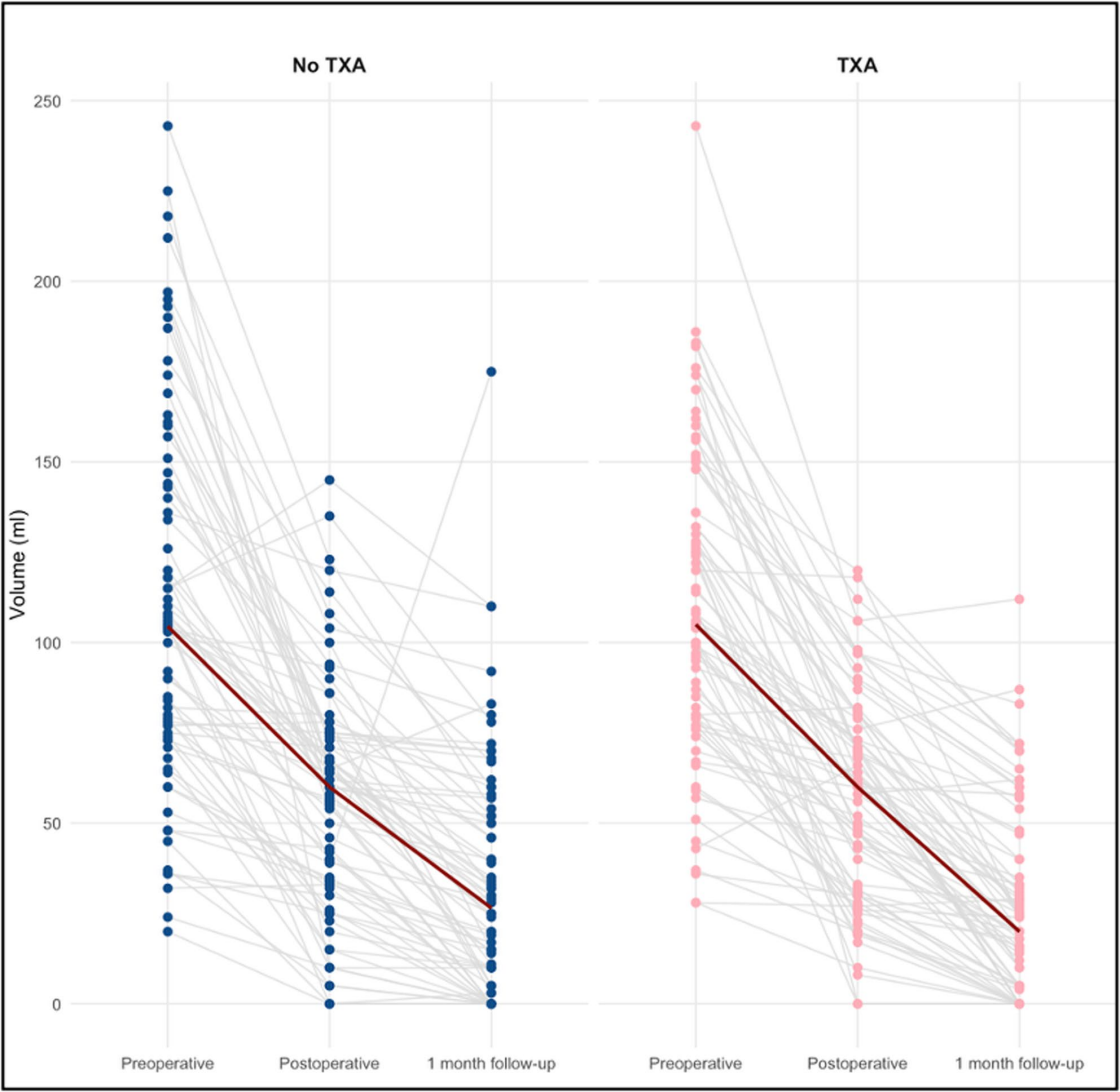
Volume development from preoperatively to a one-month follow-up; the red line represents the median volume development per treatment arm. TXA: tranexamic acid

Similarly, absolute hematoma volume reduction from baseline to one-month follow-up did not differ significantly between the TXA and control groups (85 mL vs. 68 mL; *p* = 0.361). (Fig. 4)

Receiver operating characteristic (ROC) analysis identified postoperative residual hematoma volume as a moderate predictor of revision surgery (area under the curve [AUC] = 0.683). The optimal cutoff value was determined by maximizing the Youden index, defined as sensitivity plus specificity minus one, and was *103.5 mL.* Residual hematoma volumes above this threshold were associated with an increased risk of revision surgery. In a threshold-based event analysis, the absolute proportion of clinically relevant residual hematomas (> 103.5 mL) was consistently lower in the TXA group. Postoperatively, 4/73 patients (5.5%) in the TXA group compared with 8/73 (11.0%) in the non-TXA group exceeded the cutoff (*p* = 0.533). At 1-month follow-up, residual hematoma volumes above the threshold were observed in 1/73 patients (1.4%) receiving TXA and 2/73 (2.7%) without TXA (*p* = 1.000).

## Discussion

After propensity score matching, the study cohorts demonstrated good balance with respect to baseline characteristics. Age distribution and sex ratios were comparable between groups and consistent with those reported in similar studies [2, 15, 24, 27, 31, 32, 34, 35], allowing for a valid comparative analysis both between cohorts and within the context of the current literature.

Postoperative TXA administration was associated with a lower recurrence rate following surgical evacuation of chronic subdural hematoma. However, the corresponding 95% confidence interval (0.14–1.12) marginally crossed unity, indicating limited precision and statistical uncertainty. Although the point estimate favored TXA, the findings should therefore be interpreted cautiously and regarded as hypothesis-generating rather than definitive evidence of efficacy. The recurrence rate observed in the control group (19.2%) is consistent with previous reports of standard surgical treatment [17, 22]. The magnitude and direction of the observed effect align with studies reporting reduced recurrence under TXA administration [15, 27, 31, 34], while other investigations have yielded neutral or conflicting results [2, 24, 32]. Thus, our findings contribute to an evolving and heterogeneous body of evidence.

With regard to TXA dosing, 61.6% of patients in the TXA group received a total daily dose of 1,500 mg. Notably, 66.7% of patients in the TXA group who required revision surgery had received daily doses below 1,500 mg (500 mg/day: 16.7%; 750 mg/day: 50%). Current literature does not provide a clear dose recommendation for TXA in the treatment of cSDH, with reported daily doses ranging from 500 to 1,500 mg [2, 15, 27, 31, 32, 34, 35]. Only the studies by Xie et al. [31] and Workewych et al. [32] administered a uniform dose of 1,500 mg/day, and among these, only Xie et al. demonstrated a significant reduction in recurrence rates. Consequently, neither the present study nor the existing literature allows for definitive conclusions regarding a dose–response relationship between TXA and recurrence reduction, underscoring the need for further investigation.

Anticoagulation was explicitly included as a matching variable and was perfectly balanced in the matched cohort. A sensitivity analysis excluding all anticoagulated patients demonstrated a comparable effect estimate, although statistical significance was not retained, likely due to reduced sample size. These findings suggest that the observed association is unlikely to be solely driven by anticoagulation imbalance. Methodological limitations related to anticoagulation management, including the lack of standardized documentation regarding timing of postoperative reinitiation, are discussed in the Limitations section.

No increase in mortality was observed in the TXA group. Although the retrospective nature of the study and incomplete documentation precluded systematic assessment of thromboembolic adverse events, no deaths occurred in the TXA group during the three-month follow-up period. In contrast, Salim et al. [24] reported increased mortality under TXA, attributing this finding to prothrombotic mechanisms combined with interruption of anticoagulation therapy. In our matched cohort, comorbidities and anticoagulation status were balanced, and no signal toward increased mortality was detected. In the broader context, several studies suggest that the use of TXA in neurosurgical procedures is not associated with an increased risk of thromboembolic events. This is supported by a meta-analysis by Xiong et al. [33] and a systematic review by Brown et al. [1], both of which found no significant increase in thromboembolic complications. Similarly, the TICH-2 trial reported no increase in thromboembolic events associated with TXA use in hyperacute spontaneous intracerebral hemorrhage [29].

With regard to radiological volume evolution, no significant differences in short-term hematoma volume trajectories were observed between groups. These findings are consistent with systematic reviews and meta-analyses suggesting that TXA may reduce recurrence without necessarily accelerating early volumetric resolution. In the meta-analysis by Musmar et al., TXA was associated with a significant reduction in recurrence rates and with smaller hematoma volumes during longer-term follow-up, particularly at three months, while short-term postoperative volumetric differences were less consistent [18]. Similarly, Mishra et al. reported a trend toward earlier and more complete hematoma resolution under TXA; however, these findings were insufficient to support routine TXA administration based solely on volumetric outcomes [14]. Comparable conclusions have been reported by Messias et al., who demonstrated a significant reduction in recurrence rates with TXA while observing inconsistent effects on radiological volume evolution, supporting the notion that the clinical benefit of TXA is not primarily mediated through accelerated hematoma resorption [12].

The ROC analysis suggests that recurrence risk may be threshold-dependent rather than linearly associated with residual volume. While median residual hematoma volumes did not differ between groups, there was a—albeit not statistically significant—difference in the absolute proportion of clinically relevant large residual volumes. Postoperatively, more patients in the control group exceeded the predefined threshold compared with the TXA group (8 vs. 4 cases), a pattern that persisted at the 1-month follow-up (2 vs. 1 case). The ROC analysis identified a cutoff of approximately 103.5 mL as a threshold associated with an increased risk of revision surgery. In this context, TXA does not appear to induce a global reduction in median hematoma volume but rather reduces the number of patients reaching a volume-based high-risk constellation.

From a pathophysiological perspective, recurrence of cSDH is driven by persistent microhemorrhage, inflammatory activity, angiogenesis, and hyperfibrinolysis within the hematoma membranes [3, 7]. The observed reduction in recurrence rates under TXA despite comparable volume trajectories may indicate that TXA primarily modulates these biological processes, for example by stabilizing the postoperative hematoma bed and inhibiting local fibrinolytic activity, rather than by promoting direct hematoma resorption. However, this mechanistic interpretation remains speculative and cannot be causally established based on the present data. These findings are in line with previous clinical studies, including the randomized open-label study by Wan et al. [31] and the prospective study by Yamada et al. [34], both of which reported lower recurrence rates under TXA without evidence of accelerated hematoma resorption.

The median time to revision surgery was shorter in the TXA group (8 days, range: 8–14 days) compared with the control group (11 days, range: 1–91 days). Although this observation is limited by the small number of recurrence events (*n* = 6 vs. *n* = 14), the narrow range in the TXA group suggests that recurrences under TXA, when they occur, may cluster within the early postoperative period. One possible interpretation is that TXA effectively prevents late recurrences driven by membrane-associated hyperfibrinolysis and inflammatory remodeling, while early recurrences—potentially related to surgical factors such as residual hematoma, incomplete brain re-expansion, or acute re-bleeding—may be less amenable to antifibrinolytic modulation. However, this interpretation remains speculative given the small sample size.

The role of TXA should also be considered in the context of emerging adjunctive treatment strategies. Several recent meta-analyses of randomized controlled trials have demonstrated that adjunctive or perioperative embolization of the middle meningeal artery (MMAE) is associated with a significant reduction in recurrence rates following surgical evacuation of cSDH [16, 26]. The recurrence rates reported in these studies appear to be in a range comparable to the low revision rates observed under TXA in the present investigation. It can therefore be hypothesized that adjuvant TXA administration may reduce recurrence rates to a level that has thus far primarily been achieved through post-surgical MMAE. Should this assumption be confirmed, TXA would represent an adjuvant therapeutic option that is potentially less invasive, more widely available, and more cost-effective than endovascular embolization. However, due to differences in study design, patient populations, and endpoints, direct comparability of results is currently not justified. Such comparative evaluation requires prospective randomized studies. In this regard, the currently recruiting multicenter TABASCO trial [25] represents a promising opportunity to systematically investigate the efficacy of TXA in direct comparison with MMAE as an adjuvant treatment strategy.

In summary, this matched retrospective analysis demonstrates a consistent association between postoperative TXA administration and lower recurrence rates following cSDH evacuation, despite comparable median residual hematoma volume evolution. These findings suggest that recurrence development is not solely determined by short-term volume reduction but is influenced by exceeding a critical residual volume threshold as well as underlying biological processes. However, given the width of the confidence interval and the limitations inherent to the study design, definitive conclusions regarding efficacy cannot be drawn. Whether TXA exerts its potential protective effect primarily through modulation of local hemostatic or inflammatory mechanisms, and whether it may represent a less invasive alternative to interventional adjunctive procedures, should be addressed in future prospective, ideally randomized studies.

### Limitations

This study has several methodological limitations. First, as a retrospective analysis, it is inherently susceptible to systematic bias, including residual confounding despite the use of propensity score matching. Although matching achieved good balance across key revision-associated covariates—such as age, hematoma architecture and baseline volume, anticoagulant use, arterial hypertension, and hematoma localization—unmeasured factors, including overall comorbidity burden or disease course, may still have differed between groups.

Anticoagulation represents a well-established risk factor for recurrence. While anticoagulation status was explicitly included as a matching variable and was perfectly balanced in the matched cohort, detailed and standardized documentation regarding the timing of postoperative reinitiation of anticoagulant therapy was not consistently available. Consequently, potential differences in anticoagulation resumption strategies between groups could not be formally controlled for and may have influenced recurrence risk. In an additional sensitivity analysis excluding all patients receiving oral anticoagulation therapy, the direction and magnitude of the association between TXA and reduced recurrence remained comparable; however, statistical significance was not retained, most likely due to reduced sample size. These findings suggest that the observed association is unlikely to be solely driven by anticoagulation status, yet residual confounding related to anticoagulation management cannot be fully excluded.

Second, the primary effect estimate was associated with a relatively wide 95% confidence interval that marginally crossed unity, indicating limited precision. Although the point estimate favored TXA, the study was not powered to provide definitive evidence of efficacy, and the results should therefore be interpreted with caution.

Third, neither patient adherence to TXA therapy nor standardized dosing across the cohort could be ensured, substantially limiting interpretation of potential dose–response relationships. TXA administration was not governed by a predefined institutional protocol, and daily doses varied between 500 and 2,000 mg.

Furthermore, the decision to administer TXA was left to the discretion of the treating neurosurgeon without predefined clinical criteria, and the rationale for individual treatment decisions was not documented. This introduces a risk of indication bias, as systematic differences between treated and untreated patients beyond the matched covariates cannot be excluded. A total of ten neurosurgeons were involved in patient management during the study period, and potential surgeon-level variability in treatment preferences could not be formally assessed retrospectively.

Fourth, the overall follow-up period of three months restricts conclusions regarding long-term outcomes, including mortality and late recurrence. Radiological follow-up was limited to one month postoperatively due to incomplete imaging data beyond this time point. Although no deaths occurred in the TXA group, systematic data on thromboembolic events were unavailable, precluding formal safety assessment.

Methodologically, propensity score matching is associated with loss of statistical power due to exclusion of unmatched patients, reducing effective sample size, even though complete matching was achieved for the TXA cohort. Finally, propensity score methods account only for observed covariates; unmeasured confounders influencing both treatment allocation and outcomes may persist, and residual bias cannot be excluded.

## Conclusion

In this propensity score–matched analysis, postoperative tranexamic acid was associated with a lower rate of revision surgery following chronic subdural hematoma evacuation. However, the corresponding 95% confidence interval was wide and marginally crossed unity, indicating limited precision and statistical uncertainty. These findings should therefore not be interpreted as definitive evidence of efficacy. No increase in mortality was observed. Although overall hematoma volume reduction did not differ significantly between groups, TXA-treated patients less frequently exceeded a clinically relevant residual volume threshold. Given the retrospective design and limited statistical power, the results are hypothesis-generating and require confirmation in adequately powered prospective randomized trials.

## Data Availability

Relevant data and materials are available by contacting the corresponding authors upon reasonable request.

## Abbreviation

AUC: Area under the curve
CI: Confidence intervals
CKD: Chronic kidney disease
cSDH: Chronic subdural hematoma
CT: Computed tomography
DOAC: Direct oral anticoagulant
ml: milliliter
MMAE: Middle meningeal artery embolization
OR: Odds ratio
PAI: Platelet aggregation inhibition
presur.: presurgical
PSM: Propensity Score Matching
ROC: Receiver operating characteristic
TXA: Tranexamic acid
